# Unmanned Aerial Vehicle (UAV) Imagery for Plant Communities: Optimizing Visible Light Vegetation Index to Extract Multi-Species Coverage

**DOI:** 10.3390/plants14111677

**Published:** 2025-05-30

**Authors:** Meng Wang, Zhuoran Zhang, Rui Gao, Junyong Zhang, Wenjie Feng

**Affiliations:** Shandong Academy of Agricultural Sciences, Jinan 250100, China; wangmeng_t39n@saas.ac.cn (M.W.); zhangzhuoran@saas.ac.cn (Z.Z.); gaorui_3ouy@saas.ac.cn (R.G.); zhangjunyong@saas.ac.cn (J.Z.)

**Keywords:** vegetation, UAV, vegetation index, coverage extraction, plant community

## Abstract

Low-cost unmanned aerial vehicle (UAV) visible light remote sensing provides new opportunities for plant community monitoring, but its practical deployment in different ecosystems is still limited by the lack of standardized vegetation index (VI) optimization for multi-species coverage extraction. This study developed a universal method integrating four VIs—Excess Green Index (EXG), Visible Band Difference Vegetation Index (VDVI), Red-Green Ratio Index (RGRI), and Red-Green-Blue Vegetation Index (RGBVI)—to bridge UAV imagery with plant communities. By combining spectral separability analysis with machine learning (SVM), we established dynamic thresholds applicable to crops, trees, and shrubs, achieving cross-species compatibility without multispectral data. The results showed that all VIs achieved robust vegetation/non-vegetation discrimination (Kappa > 0.84), with VDVI being more suitable for distinguishing vegetation from non-vegetation. The overall classification accuracy for different vegetation types exceeded 92.68%, indicating that the accuracy is considerable. Crop coverage extraction showed a minimum segmentation error of 0.63, significantly lower than that of other vegetation types. These advances enable high-resolution vegetation monitoring, supporting biodiversity assessment and ecosystem service quantification. Our research findings track the impact of plant communities on the ecological environment and promote the application of UAVs in ecological restoration and precision agriculture.

## 1. Introduction

Vegetation is the most important component of terrestrial ecosystems, playing a significant role in maintaining ecosystem balance, conserving water sources, and preserving soil and water [1]. Vegetation coverage is a crucial parameter for depicting the distribution of surface vegetation. It reflects a crop’s capacity to intercept light and, when considering factors influencing crop distribution and evaluating the regional ecological environment, serves as an important metric for indicating crop growth, development, and biological yield [2,3]. It is commonly defined as the proportion of the vertical projection area of vegetation to the total unit area [4].

The extraction of vegetation coverage mainly relies on ground measurement and remote sensing information [5,6,7]. Ground measurement includes visual inspection, sampling, and photography [8]. Ground measurement is limited by human factors and has low accuracy, making it difficult to obtain vegetation coverage at the regional scale. It has gradually evolved into an important means of verifying remote sensing inversion results. The methods for remote sensing inversion of vegetation coverage include color space, vegetation indices, and machine learning classification methods, which are currently the main methods applied for remote sensing estimation of vegetation coverage [6].

Amidst the swift advancement of science and technology, unmanned aerial vehicles (UAVs) have become increasingly mature and represent an important remote sensing platform [9]. In recent years, UAV remote sensing has witnessed rapid development. It leverages advanced unmanned aerial flight technology to quickly acquire spatial information about land, resources, and the environment. Compared with traditional satellite remote sensing and aerial remote sensing, the technology has advantages such as high spatial accuracy and low cost. Currently, it has been widely used in fields such as surveying, disaster monitoring, hydro-meteorology, and resource investigation [10,11,12,13].

Researchers have extensively investigated various methods for extracting vegetation coverage based on UAV remote sensing technology [14,15,16]. Extracting vegetation information using vegetation indices was an important indicator for evaluating vegetation growth status and was also one of the most commonly used methods in vegetation monitoring [17,18]. The commonly used indices in vegetation coverage research using UAV are mainly divided into visible light and visible light–near infrared vegetation indices [19]. The visible light vegetation index was developed utilizing the prominent reflection properties of green vegetation in the green light spectrum and the absorption characteristics towards red and blue light [20]. Scholars have constructed various typical visible light vegetation indices based on these characteristics, such as the normalized green-red difference index (NGRDI), visible light band difference vegetation index (VDVI), green-red vegetation index (GRVI), green-blue vegetation index (RGBVI), and over green index (EXG). There are over 100 visible light–near infrared plant cover indices, such as the normalized difference vegetation index (NDVI), soil-adjusted vegetation index (SAVI), and enhanced vegetation index (EVI) [21,22,23]. Currently, these indices are widely used in the research of UAV multispectral remote sensing vegetation parameter inversion [20]. For example, J. Zhao et al. [24] used a UAV to capture visible light images of corn fields. They then employed a combination of supervised classification and statistical histograms of visible light vegetation indices to determine the threshold for extracting corn vegetation coverage. Niu et al. [25] verified that UAV multispectral remote sensing can effectively extract winter wheat vegetation coverage information, and they concluded that compared with SAVI and the modified soil-adjusted vegetation index (MSAVI), the extraction effect based on the NDVI classification threshold was the best. Jay et al. [26] found that compared with NDVI, the chlorophyll red edge index (CIred), chlorophyll green band index (CIgreen), medium-resolution imaging spectrometer (MERIS) chlorophyll index (MTCI), and visible light impedance vegetation index (VARI) can suppress the impact of differences in lighting conditions and effectively extract vegetation coverage. Choi et al. [14] used a fixed-wing UAV to obtain multispectral images and estimated partial vegetation coverage on sand dunes. Li et al. [15] proposed a method to obtain vegetation index thresholds using winter wheat as an example. Wang et al. [27] constructed the vegetation index VDVI and proposed a bimodal histogram threshold method and histogram entropy threshold method to obtain vegetation index thresholds. Bendig et al. [28] used a crop surface height model based on a UAV to monitor barley biomass using visible light and near-red cover indices. Kyratzis et al. [29] used a UAV to monitor barley biomass. The vegetation index of wheat crop phenotype in the Mediterranean region was effectively evaluated using images. Li et al. [30] took the riparian zone of the middle and lower reaches of the Yangtze River as an example and conducted research on the point cloud vegetation filtering algorithm for UAV LiDAR.

The similarity of the above studies lies in the use of UAV remote sensing images to explore a suitable method for extracting vegetation indices for specific vegetation, especially their focus on single plant species and dependence on multispectral data, which have limitations regarding the convenient monitoring of multi-species communities. So far, there have been hundreds of vegetation indices proposed both domestically and internationally. However, the majority of these indices incorporate both visible light and near-infrared bands [31,32,33,34]. At present, low-cost consumer-grade UAVs, typically equipped with RGB cameras, are more commonly used in practical applications. Considering factors such as the difficulty and cost of image acquisition, the use of visible light band UAV images is more widespread. Therefore, drawing on existing research results, selecting representative visible light vegetation indices, and exploring simple, applicable, and effective vegetation coverage extraction methods based on UAV visible light images represent important areas for scientific questioning.

In order to accurately quantify the canopy coverage of multi-vegetation, this study focused on the typical vegetation types (crops, trees, and shrubs) in the North China Plain, aiming to establish a robust framework for vegetation coverage extraction. By integrating visible light vegetation index and machine learning-based threshold optimization, we have developed a low-cost, operator-agnostic method that can handle complex plant community cover. This verification method significantly improves the applicability of UAV remote sensing in high-precision vegetation inversion while providing a scalable solution for ecological protection and plant community monitoring under environmental gradients.

## 2. Materials and Methods

### 2.1. Study Area

**Figure 1:**
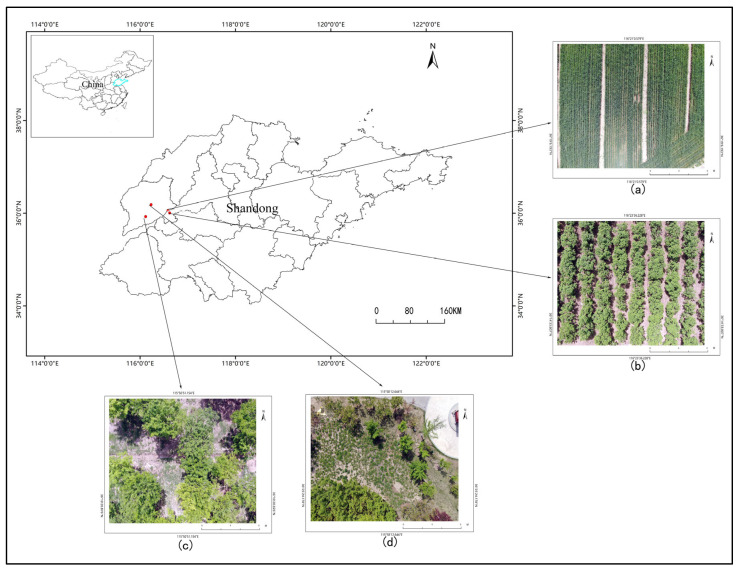
Overview of the study area. (**a**) Crops in Dong’a County; (**b**) shrubs in Pingyin County; (**c**) trees in Yanggu County; (**d**) mixed vegetation in Dongchangfu District.

The study area is located in the western part of Shandong Province, China, and in the northwest of the North China Plain. It experiences a continental climate within the East Asian monsoon region of the North Temperate Zone, characterized by dry springs, hot and rainy summers, cool autumns, and long, cold winters. In order to conduct research on the extraction of coverage information of various vegetation based on visible light images of UAV, this study obtained visible light image data of crop (wheat), tree (beech), shrub (roses), and mixed vegetation (including tree, shrub, and natural grasslands) using a UAV. The experimental areas are located in Dong’a County, Yanggu County, Pingyin County, and Dongchangfu District. An overview of the study area is shown in Figure 1.

### 2.2. UAV Image Data Acquisition

The experiment used a DJI Mavic 2 UAV (DJI, Shenzhen, China) with a focal length of 24 mm; the spectral range of the visible light lens it is equipped with is shown in Table 1. The data collection and shooting time were on 12 April 2024, and all data were collected between 10:00 and 12:00 (Beijing time) on that day when the weather was sunny. Four sites, which were in Dong’a County, Yanggu County, Pingyin County, and Dongchangfu District, were selected as experimental areas, with their center point coordinates being 116°20′56″ E/36°18′44″ N, 115°50′51″ E/36°10′48″ N, 116°22′52″ E/36°15′12″ N, and 115°58′8″ E/36°25′22″ N. Different UAV flight heights were set up in the same experimental area, and images of different spatial resolutions (0.83 cm, 1.25 cm, 1.67 cm, and 2.08 cm) were obtained. The UAV hovered for shooting, and the weather conditions were good during the shooting, with no wind or clouds. The UAV mission captured 192 visible light images throughout the entire study area. To ensure high-precision orthorectified mosaic generation and minimize geometric distortion, the flight plan was configured with 85% side panels and 85% front panels. This overlapping design promotes robust image stitching and minimizes feature displacement, which is crucial for maintaining spatial consistency in vegetation cover extraction. The weather conditions during the period when the UAV obtained visible light images of crop, tree, shrub, and mixed vegetation were consistent. This study ignored the influence of weather on the visible light image processing results, such as cloud cover and diffuse light, as aerial photography is limited to clear sky periods to standardize lighting conditions and simplify data processing workflows. This study utilized Pix4DMapper software (v.4.4.9, Pix4D SA, Lausanne, Switzerland) for image stitching and orthophoto generation. The software systematically processed UAV imagery through automated steps, including image alignment, georeferencing, and orthorectification. The Ag RGB Rapid/Low Res template was specifically selected to balance processing speed and vegetation-specific accuracy, optimizing for fast orthomosaic generation in agricultural and ecological applications. This template streamlines workflow by prioritizing efficient feature detection in visible light bands while reducing computational load through adaptive resolution sampling. The resulting orthophoto maps were georeferenced to the WGS 84 coordinate system, ensuring spatial consistency with ground reference data and enabling precise vegetation coverage mapping across experimental areas. In order to protect data integrity, point clouds used standard precision mode to ensure transmission security while maintaining geometric accuracy. We applied adaptive correction to UAV images to reduce brightness differences caused by variable lighting. In this study, there were no strict requirements for the position and band range of the center wavelengths of visible light in the red, green, and blue bands, and the obtained images were not radiometrically calibrated.

### 2.3. Methods

**Figure 2:**
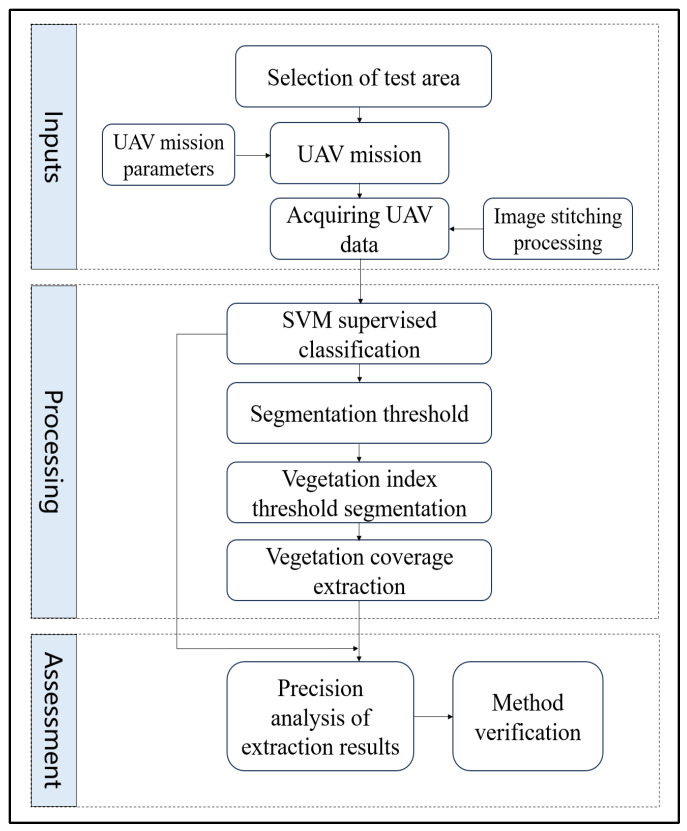
Flow chart of vegetation coverage extraction.

The vegetation coverage extraction methods based on remote sensing technology can be mainly divided into the empirical model, physical model, pixel decomposition, and vegetation index threshold methods [6,15]. The empirical model method is only applicable to specific regions and vegetation types, while the physical model method requires a large amount of data. The pixel decomposition method also has certain accuracy issues, which limit the application of the above methods. Based on the corresponding vegetation index threshold, vegetation coverage extraction is feasible, and the analysis results are reliable [15,35,36]. It has good application prospects in large-scale and multi-vegetation coverage measurement. In this study, single visible light images of crops, trees, and shrubs were selected for the experiment. Envi 5.3 software was used to supervise the classification of the three vegetation types using SVM. The EXG, VDVI, RGRI, and RGBVI values of vegetation and soil were used as the horizontal axis, and the number of pixels was used as the vertical axis to draw statistical histograms of vegetation and soil pixels. The intersection area of vegetation pixels and non-vegetation pixels in the statistical histogram was used as the threshold area for vegetation index segmentation. Suitable image segmentation thresholds were selected, and vegetation coverage extraction was performed using the EXG, VDVI, RGRI, and RGBVI vegetation indices. The visible light image of the UAV was segmented into vegetation and non-vegetation for vegetation coverage extraction, and we analyzed and verified the extraction results. The main process of the extraction method is shown in Figure 2.

#### 2.3.1. Selection of Vegetation Index

A vegetation index is a method used to quantify vegetation characteristics by leveraging the reflective and absorptive properties of green vegetation in response to electromagnetic waves. This is achieved by combining sensitive spectral bands either linearly or nonlinearly. In the field of remote sensing applications, vegetation indices have been extensively employed for qualitative and quantitative evaluations of vegetation coverage and its growth vitality. Because of the intricate interplay of vegetation, soil reflectance, environmental influences, shading effects, and soil hue and humidity in vegetation spectra, and due to the influence of atmospheric spatial and temporal changes, there is no universal value for the vegetation index, and its research often shows different results.

The commonly used vegetation indices in current research applications include the normalized vegetation index, the ratio vegetation index, the difference vegetation index, the vertical vegetation index, the orthogonal value vegetation index, etc. According to the type of UAV remote sensing data, studies have shown that using UAVs to extract vegetation indices commonly used in vegetation coverage research can be categorized into two groups: visible light vegetation indices and visible light-near infrared vegetation indices. The construction of the visible light vegetation index was based on the strong reflection characteristics of green vegetation and the absorption characteristics of red and blue light. Guided by the demand for consumer-grade drones without near-infrared sensors and for distinguishing complex vegetation types, we have selected four VI models that have been validated for performance in only RGB workflows:

- EXG [27] enhances the contrast between green vegetation and non-vegetative backgrounds (e.g., soil and dry matter) by leveraging the strong green reflectance of live plants, which is critical for initial vegetation/non-vegetation screening;
- VDVI [27] minimizes soil brightness interference by normalizing red-green band differences, a known challenge in heterogeneous landscapes where soil variability can skew classification accuracy;
- RGRI [37] was included for its simplicity and effectiveness in distinguishing structural differences (e.g., dense crops vs. sparse shrubs) through the red-green spectral ratio, which correlates with leaf chlorophyll content;
- RGBVI [38] incorporates all three visible bands to capture subtle spectral variations between vegetation types, as demonstrated in prior studies where it outperformed single-band indices in mixed plant communities.

Unlike NIR-dependent indices (e.g., NDVI), these VIs operate exclusively on RGB channels, aligning with the hardware constraints of low-cost UAVs (e.g., DJI Mavic 2 used in this study) and enabling accessible, scalable vegetation monitoring. The calculation process of these four vegetation indices is as follows:

(1) $$\mathrm{EXG}=2\rho_{g}-\rho_{r}-\rho_{b}$$

(2) $$\mathrm{VDVI}=\frac{2\rho_{g}-\rho_{r}-\rho_{b}}{2\rho_{g}+\rho_{r}+\rho_{b}}$$

(3) $$\mathrm{RGRI}=\frac{\rho_{r}}{\rho_{g}}$$

(4) $$\mathrm{RGBVI}=\frac{{\rho_{g}}^{2}-\rho_{r}{*\rho }_{b}}{{\rho_{g}}^{2}+\rho_{r}{*\rho }_{b}}$$

$\rho_{r}$, $\rho_{g}$, and $\rho_{b}$ represent the pixel values of visible light in the red, green, and blue bands, respectively.

#### 2.3.2. SVM

SVM is a machine learning algorithm based on the principles of statistical learning theory. Its fundamental model is a linear classifier that is defined by the largest margin within the feature space. It includes core techniques, which make it essentially a nonlinear classifier [39], and belongs to supervised classification. The algorithm takes those error-prone and problematic sample points as the support points of the classification surface and optimizes the classification discrimination surface to maximize the interval between positive and negative support surfaces. For the training sample set, it can select a small number of sample points as base vectors to design classifiers, reducing testing time. For limited training sample sets in high-dimensional feature spaces, it can generate generalized classifiers, effectively solving the problem of small samples.

To verify the accuracy of the supervised classification method of SVM, this study adopted visible light remote sensing images with a spatial resolution of 0.83 cm in crop experimental areas as an example. Combined with the results of the field ground investigation, 100 vegetation and 100 soil samples in this area were selected on the image based on photo interpretation for SVM-supervised classification. A total of 100 vegetation and 100 soil samples were spatially independent of the training data and were only used for validation. The position and category of each ground truth sample were compared with the SVM classification results to calculate the confusion matrix. The confusion matrix of the classification results of vegetation and soil in the crop experimental area was calculated for accuracy verification, as shown in Table 2, and the Kappa coefficient was calculated to be 0.98, indicating that the supervised classification results of SVM have high accuracy.

Based on the above experiments and considering the practicality of the experimental data and the convenience of the software program, this study uses SVM-supervised classification to distinguish between vegetation and non-vegetation pixels.

#### 2.3.3. Method for Determination of Vegetation Index Threshold

The vegetation index threshold method was an effective approach for differentiating between vegetation and non-vegetation. After calculating the vegetation index, the histogram threshold method was employed to establish a threshold value. Pixels with a vegetation index higher than this threshold were classified as vegetation, while those with a value lower than the threshold were categorized as non-vegetation [15,18].

#### 2.3.4. Correlation Analysis of UAV Image Resolution

This method evaluates the impact of spatial resolution on the accuracy of vegetation cover extraction by systematically analyzing multi-resolution visible light UAV images. It involves capturing UAV images of the same area at multiple spatial resolutions under the same conditions; applying a fixed visible vegetation index threshold to extract vegetation coverage at all image resolutions; and comparing the vegetation cover estimation based on vegetation index threshold classification with the SVM-derived results to quantify the accuracy changes related to spatial resolution. By using image resolution as the independent variable, this method aims to explore the optimal UAV image resolution for vegetation monitoring. This method provides support for UAV-based remote sensing decision-making by revealing how UAV image resolution affects feature recognition in visible light images.

### 2.4. Accuracy Verification of Vegetation Coverage Extraction Results

The common method for evaluating the precision of vegetation coverage extraction is to obtain the area of interest through photo interpretation for evaluation; this is based on its vegetation canopy coverage, combined with ground survey data and image reality for photo interpretation. However, this method is limited by human and material resources and is not suitable for evaluating the extraction results of vegetation coverage in multi-vegetation and large area regions.

At present, the main method for verifying the accuracy of vegetation coverage extraction is to use the images collected by field photography as the true values and verify the results of vegetation coverage extraction through photo interpretation. Due to the limitations of human and material resources, field photography is not suitable for verifying the accuracy of large-scale vegetation coverage extraction. With the development of machine learning and remote sensing technology, using supervised classification results as the true value of vegetation coverage has achieved good results in verifying the accuracy of vegetation coverage extraction. In order to balance practicality and scalability, considering the challenge of obtaining large-scale ground truth through field investigations (such as labor-intensive GPS reference map sampling), SVM-supervised classification results trained on visible light images of UAVs are used as approximate true values for method validation. This method provides a relatively accurate assessment but does not represent the absolute ground truth in the strictest sense. The extraction error is the difference between the vegetation coverage value obtained by the vegetation index threshold method and the value obtained by SVM-supervised classification. The absolute error is the absolute value of the extraction error divided by the percentage of the outcome value obtained from SVM-supervised classification.

## 3. Results

### 3.1. Determination of Vegetation Index Threshold

**Figure 3:**
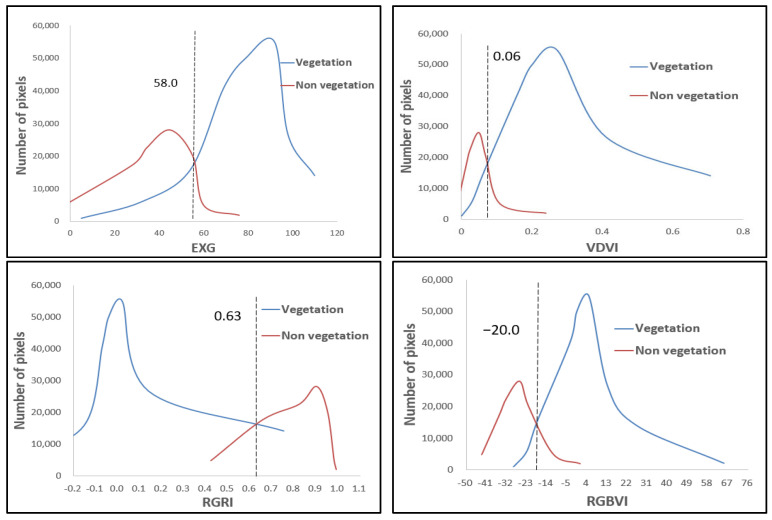
Histogram of vegetation index statistics for crop.

**Figure 4:**
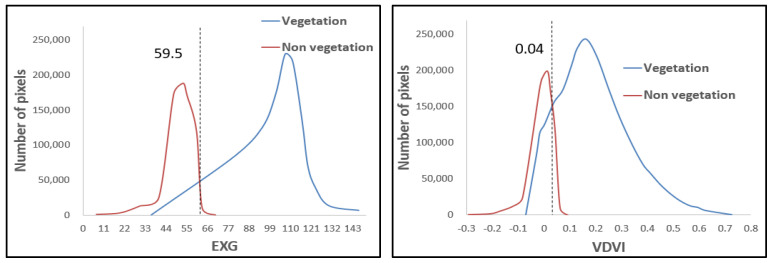

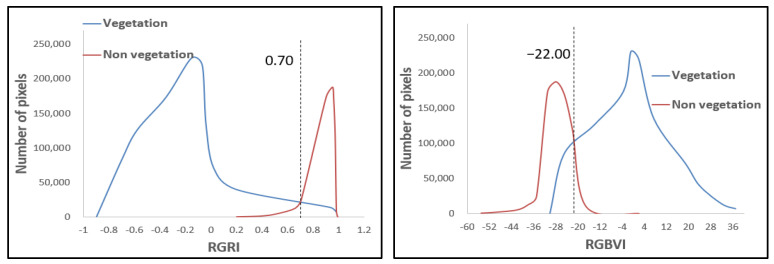
Histogram of vegetation index Statistics for tree.

**Figure 5:**
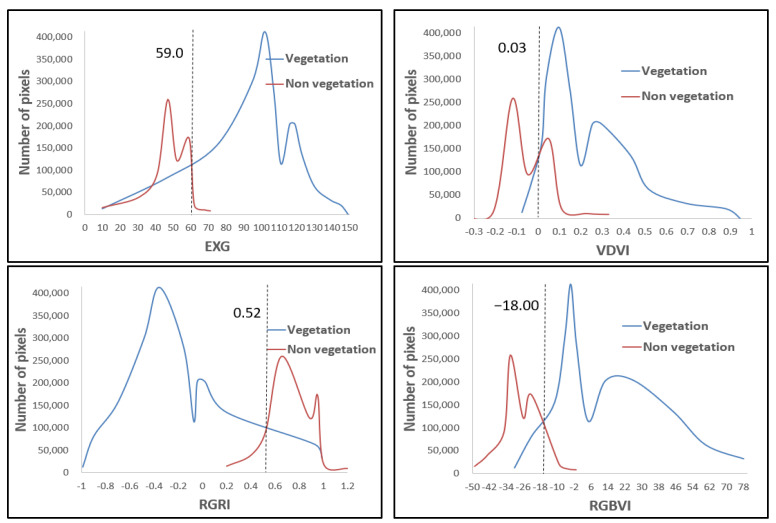
Statistical histogram of vegetation indices for shrub.

Based on the supervised classification results, statistical analysis was conducted on four vegetation indices (EXG, VDVI, RGRI, and RGBVI) from visible light images of a UAV to quantify the spectral separability between vegetation (crops, trees, and shrubs) and non-vegetation (mainly soil) pixels. Figure 3, Figure 4 and Figure 5 show the statistical histograms of each vegetation index, where the double peaks represent the different spectral characteristics of vegetation and soil.

As shown in Figure 3, the statistical histograms of crop vegetation and soil pixels display a high–low bimodal distribution for the selected vegetation indices. The vegetation histogram curve is more detailed than the soil histogram curve, indicating that the number of crop vegetation pixels in the experimental area is significantly higher than that of soil pixels. As shown in Figure 4, the statistical histograms of the selected vegetation indices for tree vegetation and soil pixels also exhibit a high–low bimodal distribution. The vegetation histogram curve shows a larger distribution (wider range of values) than the soil histogram curve. This wider distribution indicates that the number of tree vegetation pixels in the experimental area is significantly higher than that of soil pixels. As shown in Figure 5, the statistical histograms of the selected vegetation indices for shrub vegetation and soil pixels exhibit a bimodal distribution with high and low values, and the histogram curves are close, indicating that the number of shrub vegetation pixels in the experimental area is slightly higher than that of soil pixels. As shown in Figure 3, Figure 4 and Figure 5, the intersection points of the statistical histograms of vegetation and soil pixels in EXG are 58.00, 59.50, and 59.00, respectively. For VDVI, these intersection points are 0.06, 0.04, and 0.03, respectively. In the case of RGRI, the intersection points of the corresponding histograms are 0.63, 0.70, and 0.52, respectively. Finally, for RGBVI, the intersection points of the statistical histograms of vegetation and soil pixels are −20, −22, and −18, respectively.

**Figure 6:**
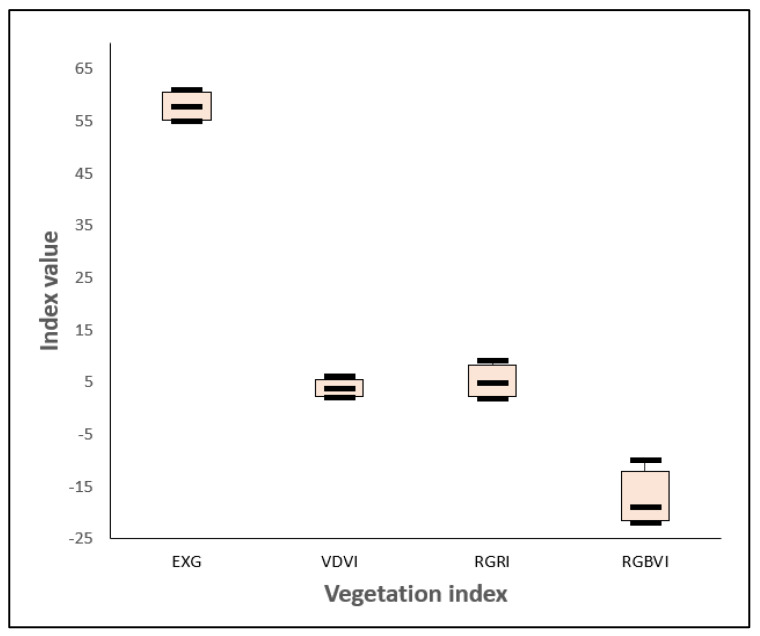
Intersection changes in vegetation and non-vegetation statistical histograms.

The optimal threshold for distinguishing between soil and vegetation using vegetation indices is determined by identifying the intersection point of the statistical histograms of soil and vegetation pixels. This method leverages the inverse relationship between vegetation growth, characterized by an increase in vegetation pixels, and soil exposure, characterized by a decrease in soil pixels, within the study area. By doing so, it ensures a dynamic, data-driven approach to threshold selection. As shown in Figure 6, the focus is on the intersection changes in vegetation and non-vegetation in the statistical histograms. The thresholds of EXG and VDVI show relatively small fluctuations among the three vegetation types (crops, trees, and shrubs), indicating higher stability compared to RGRI and RGBVI, which exhibit greater changes. This stability indicates that EXG and VDVI are less sensitive to differences in vegetation types, making them more robust in threshold determination. Overall, the changes in the intersection points of the histograms of the four vegetation indices on the three vegetation covers are relatively stable. Although there are mixed pixels in the visible light images obtained by the UAV, the intersection area of the vegetation and soil histograms is a reliable threshold range. We selected appropriate values within this range as the final segmentation threshold, balancing adaptability with spectral changes while maintaining consistency across different vegetation types. Therefore, in this study, the intersection area of the statistical histograms of vegetation pixels (for crops, trees, and shrubs) and soil pixels was selected as the threshold area for vegetation index segmentation. Appropriate values within this area were then chosen as the segmentation thresholds for differentiating vegetation from non-vegetation.

### 3.2. Extraction and Analysis of Vegetation Coverage

**Figure 7:**
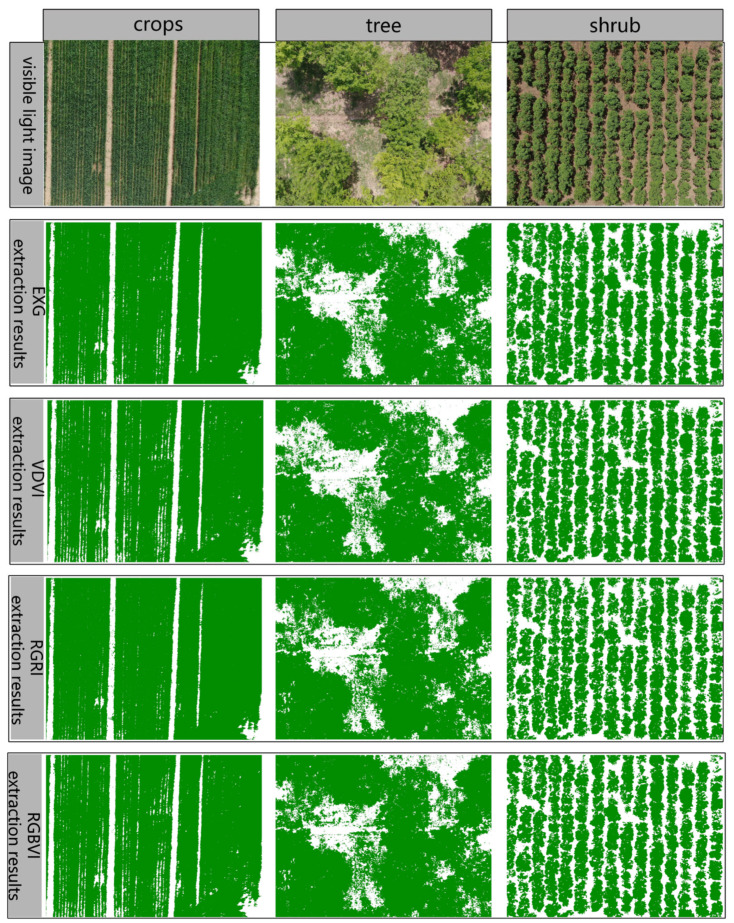
Vegetation coverage extraction results of each vegetation index.

Once the vegetation index threshold was established, the pixel distribution histograms of vegetation and soil for each vegetation index within the three vegetation cover types were separately calculated to determine the threshold area for vegetation coverage extraction. In this study, appropriate image classification thresholds were determined from the intersection of histograms in the study area. Subsequently, vegetation coverage extraction was performed on UAV-captured visible light images of crops, trees, and shrubs with a spatial resolution of 0.83 cm, as illustrated in Figure 7. To verify the accuracy of extracting vegetation coverage with different vegetation indices, combined with the ground conditions of each experimental area, vegetation points and non-vegetation points selected in each experimental area were used as truth validation data to calculate the confusion matrix. Table 3 presents the overall accuracy and Kappa coefficient for distinguishing crops, trees, and shrubs from soil using four vegetation indices.

For visible light images of crops, trees, and shrubs, threshold segmentation is performed using four vegetation indices, and the accuracy of extracting coverage varies. The overall classification accuracy for each type of vegetation exceeds 92.68%, with the highest accuracy reaching 99.93%. The results of EXG threshold segmentation indicate that the accuracies of extracting the coverage of the three vegetation types are 99.65%, 93.49%, and 99.83%, respectively. For VDVI threshold segmentation, the accuracies of extracting the coverage of the three vegetation types are 99.78%, 94.22%, and 99.92%, respectively. In the case of RGRI threshold segmentation, the accuracies are 99.78%, 92.68%, and 98.71%, respectively. Finally, the RGBVI threshold segmentation results show accuracies of 99.78%, 94.13%, and 99.93% for extracting the coverage of the three vegetation types, respectively. The main crop and shrub in the experimental area are relatively pure wheat and rose vegetation, which are easy to distinguish from the soil. The four vegetation indices threshold segmentation accuracy for crops and shrubs is generally high. However, due to the mixed presence of elm trees, weeds, and other vegetation in the experimental area, the segmentation accuracy of the four vegetation indices thresholds for trees is relatively low. Overall, the vegetation indices VDVI and RGBVI exhibit high extraction accuracy, enabling effective differentiation between vegetation and non-vegetation in the experimental area. Since soil and vegetation have overlapping reflectance in the visible light green band, distinguishing between them using only this band proves challenging. The VDVI vegetation index is constructed by fully considering the spectral characteristics of the red, green, and blue bands, which makes it particularly well-suited for differentiating vegetation from non-vegetation.

### 3.3. Extraction Results of Images with Different Spatial Resolutions

To further explore the applicability of UAV visible light images with different spatial resolutions to this method, we compared the accuracy of vegetation coverage extraction using UAV visible light images with four spatial resolutions (0.83 cm, 1.25 cm, 1.67 cm, and 2.08 cm). These images were acquired in the same region during the same period. This study chose VDVI as the fixed vegetation index and used the same vegetation index threshold extraction method to obtain vegetation coverage. For SVM, we used VDVI-based features. For each vegetation type, regardless of spatial resolution, the same SVM settings were applied, and the average SVM-supervised classification results of images with different resolutions were used as the approximate true value of the vegetation coverage. The accuracy of the statistical analysis of crop, tree, and shrub vegetation coverage obtained through supervised classification and vegetation index threshold extraction is shown in Table 4.

### 3.4. Method Verification

**Figure 8:**
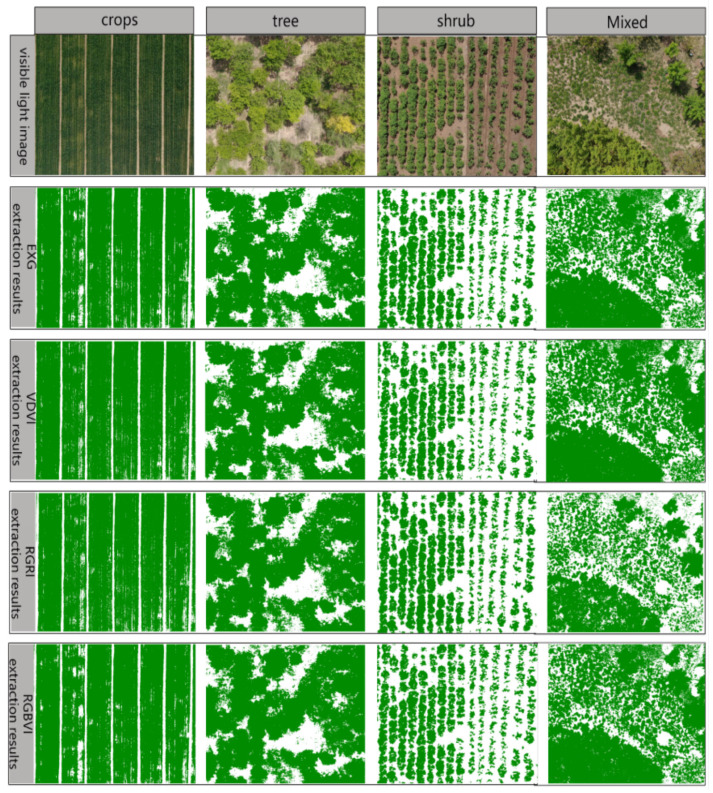
The extraction results of vegetation coverage for each vegetation index in the verification area.

In order to further verify the applicability and reliability of the vegetation coverage extraction method using UAV visible light image vegetation index threshold for crop, tree, shrub, and mixed vegetation, which includes tree, shrub, and natural grasslands, this study selected UAV visible light images with the spatial resolution 0.83 cm from different experimental areas during the same period as the data source. The vegetation coverage vegetation index threshold extracted in the previous section was used as a fixed threshold, and the same method was used for vegetation coverage extraction. The results are shown in Figure 8 to verify the applicability of the method to crops, trees, shrubs, and mixed vegetation (images with a resolution of 0.83 cm in different experimental areas during the same period). The average value of the supervised classification results by SVM was used as the true value for extracting the coverage of the three vegetation covers in this validation experiment. This means that for each vegetation type, regardless of other variables, this fixed average was used to evaluate the accuracy of the threshold extraction method, resulting in the same approximate true value. The results of the supervised classification and vegetation index threshold extraction of the vegetation coverage of crop, tree, shrub, and mixed vegetation were obtained, and their accuracy via statistical analysis is shown in Table 5.

## 4. Discussion

### 4.1. Sensitivity of Classification Threshold

Although the method of determining classification thresholds from the intersection of histograms proved effective, it is essential to consider the sensitivity of the results to minor threshold variations. To assess this, we conducted additional experiments by slightly adjusting the threshold values within a reasonable range. The results indicated that small changes in the thresholds led to minimal fluctuations in the vegetation coverage extraction results for crop, tree, and shrub visible light images. For example, when the threshold was increased or decreased by 5% of its original value, the overall classification accuracy varied by less than 2%. These findings suggest that the proposed method has relatively high robustness and that the selected threshold values can stably distinguish vegetation from non-vegetation, even with slight adjustments. However, in future research, more in-depth studies on threshold optimization across different scenarios will be conducted to further enhance the method’s adaptability.

### 4.2. The Performance of Different Indices in Extracting Different Vegetation Types

In the previous section, UAV visible light images of crops, trees, and shrubs were used to determine the segmentation threshold via statistical analysis of vegetation indices. Subsequently, vegetation coverage was extracted, and the adaptability of the method was verified. According to Table 5, except for the mixed vegetation verification area, the threshold segmentation and extraction error of each vegetation index in the crop, tree, and shrub verification areas does not exceed 5%, and the standard deviation of the error does not exceed 1.5. The extraction accuracy of EXG and VDVI is generally higher than that of RGRI and RGBVI. For different vegetation types, the accuracy of crop vegetation coverage extracted by various vegetation index threshold segmentation methods is generally higher than that of trees, shrubs, and mixed vegetation. This is because in UAV-captured crop images, vegetation and soil pixels are more uniformly distributed [24]. UAV images of trees often contain numerous mixed pixels of weeds and other vegetation. Although the distribution of vegetation and soil pixels in UAV images of shrubs is relatively uniform, a large number of vegetation shadows are also present. The threshold extraction results of various vegetation indices in the mixed vegetation verification area are the worst, with a maximum error of 5.38%, as the mixed vegetation contains a large number of mixed pixels [40,41]. The above accuracy evaluation results indicate that the overall extraction accuracy of the coverage of the three vegetation covers in the validation image is relatively high.

Research has shown that for specific vegetation, establishing a vegetation coverage estimation model and selecting EXG, VDVI, RGRI, and RGBVI can effectively extract the coverage information of the vegetation [24,25]. In this study, based on the above experimental and validation results, we conclude that the four typical UAV visible light vegetation index threshold extraction methods can be effectively used to extract vegetation information from crops, trees, and shrubs, demonstrating high extraction accuracy and overall reliability. Compared with existing UAV-based visible light image methods for vegetation coverage extraction, the approach proposed in this study offers several advantages. It can accurately extract the coverage of typical vegetation in northern China within small-scale areas and holds promising application prospects for extracting vegetation canopy information. Although the proposed method achieves high accuracy for typical vegetation in northern China, further refinement and adaptation may be necessary to accommodate different geographic regions and vegetation types. Future research will concentrate on validating the universality of our method across various ecosystems.

### 4.3. The Influence of Different Spatial Resolutions of Visible Light Images for UAV

To investigate the elasticity of spatial resolution using this method, we tested visible light images of unmanned aerial vehicles with resolutions ranging from 0.83 to 2.08 cm (Table 4). Surprisingly, the vegetation coverage extraction error remained consistently low at all resolutions (relative error ≤ 0.035, absolute error ≤ 2.5), and there was no significant correlation between resolution and accuracy. Although the error in tree coverage is slightly higher than that of crops or shrubs, which may be due to their complex crown geometry, the magnitude of the error does not increase with decreasing resolution [42]. This is in sharp contrast to traditional remote sensing methods, which typically require high-resolution images to analyze heterogeneous vegetation. The insensitivity of resolution comes from the spectral threshold framework, which utilizes consistent vegetation–non-vegetation separability across visible bands at different scales.

These findings establish a key innovation: our approach eliminates the need for expensive high-resolution sensors and enables scalable, cost-effective vegetation monitoring across different drone platforms and ecosystems.

### 4.4. Limitations and Further Work

UAV remote sensing technology has been widely studied and applied in obtaining vegetation canopy information, disease information, biomass, and other aspects due to its unique advantages, such as high spatiotemporal resolution, high work efficiency, and convenience [20,43,44]. In this study, using a UAV visible light remote sensing platform to obtain coverage information of multiple vegetation types offers significant advantages. It can rapidly acquire large-scale vegetation images, covering areas exceeding 20 hectares, thus demonstrating high work efficiency. Therefore, the proposed UAV-based visible light method serves as a valuable supplementary approach for quickly obtaining vegetation information. When integrated with broader remote sensing technologies, it can facilitate large-scale mapping efforts. Though UAV rapid coverage of over 20 ha is remarkable, scaling this method to larger areas (e.g., >100 ha) merits study. Fleet missions—where multiple UAVs operate in tandem—could sharply raise data-collection speed for vast regions, while advanced automated stitching pipelines would be vital to seamlessly merge the massive volume of captured images, ensuring accurate and comprehensive mapping. Despite inherent challenges in mission coordination and data processing, these approaches hold the potential to broaden the method’s utility in large-scale vegetation monitoring. However, despite the rapid advancement of UAV remote sensing technology, certain aspects still require improvement, such as flight stability in strong wind conditions and the accuracy of image correction under complex terrain or variable lighting scenarios.

In this research, UAV remote sensing technology was used to capture visible light imagery, focusing on diverse vegetation types and selecting appropriate vegetation indices for threshold segmentation of the visible light images. The experimental results showed that using a UAV visible light image vegetation index threshold classification method can effectively extract vegetation coverage. However, compared to areas with simple crops, trees, and shrubs, the vegetation coverage extraction method used in this study has lower accuracy in extracting mixed vegetation areas. The lower accuracy in mixed vegetation areas may be due to physiological and morphological differences between crops, trees, and shrubs. Crops such as wheat typically exhibit uniform canopy structures and spectral homogeneity in the visible spectrum, whereas shrubs and trees introduce complexity due to irregular leaf distributions, shadow effects, and spectral overlaps with soil or other vegetation. For example, shrub canopies with high stem density and small leaves may reflect light-like leaves but differ structurally, leading to classification errors when using fixed vegetation index thresholds. Future work could integrate texture analysis or multi-temporal UAV data to explain these morphological and phenological changes.

Although our method has demonstrated satisfactory performance in the North China Plain, its application to other ecosystems, such as alpine and Mediterranean regions, may require substantial adaptations. In alpine ecosystems, extreme environmental conditions, including low temperatures and high-altitude solar radiation, can alter the spectral reflectance of vegetation. This may lead to inaccuracies in vegetation coverage extraction if the existing vegetation indices and classification thresholds are directly applied. For Mediterranean ecosystems, characterized by a distinct climate with hot, dry summers and mild, wet winters and unique vegetation communities, the current method may need to be refined. The specific plant species composition and the different seasonal patterns of vegetation growth in these regions can influence the effectiveness of our method. We acknowledge that there are limits to the generalizability of our current approach, and further research is needed to optimize the method for these diverse ecosystems.

Radiation correction is the process of converting the raw digital values (DN) captured by sensors into physically meaningful radiation or reflectance values, aimed at reducing distortions caused by sensor anomalies, atmospheric attenuation, and lighting changes, ensuring that images accurately reflect the biophysical properties of the Earth’s surface [45]. Although cross-sensor universality is crucial for the visible light vegetation index derived from UAVs [46], this study omitted radiation calibration due to three background factors: (1) the data were collected using the same UAV under almost identical solar conditions, minimizing radiation variations between scenes; (2) compared with species-level spectral identification, the binary classification of vegetation/non-vegetation targets reduces sensitivity to absolute radiometric accuracy; (3) business priorities emphasize fast and cost-effective processing, consistent with the goals of quick and easy ecological monitoring work. Although the spectral range of UAV RGB sensors, especially the center wavelength and bandwidth, can affect the vegetation index threshold, empirical research has shown that when using single sensor data with consistent band parameters and obvious vegetation characteristics, this effect can be ignored for vegetation monitoring [46,47]. The validation of 100 vegetation and 100 soil samples showed high classification accuracy, confirming that strict adherence to radiometric calibration is unnecessary for binary vegetation classification based on UAVs under controlled operating conditions, provided that spectral separability between vegetation and background remains evident, as established by Bendig et al. [48] in the FAIR-compliant UAV protocol.

Overall, it can be concluded that in future research on vegetation information extraction based on visible light remote sensing data from UAVs, research is needed to investigate the impact of weather on the results of visible light image acquisition, image radiometric calibration, and atmospheric calibration of UAVs [13]. On the basis of minimizing errors in various aspects such as data sources, weather, and UAV platforms, future research needs to focus on how to integrate hyperspectral/multispectral/LiDAR platforms to enhance data acquisition capabilities and how to use deep learning remote sensing models based on multi-source data to improve the accuracy of mixed vegetation areas.

## 5. Conclusions

This study presents a low-cost, scalable framework for extracting vegetation coverage of crops, trees, shrubs, and mixed vegetation using UAV visible light imagery and vegetation index thresholding. By integrating SVM-supervised classification with spectral histogram analysis, we established robust thresholds for four vegetation indices (EXG, VDVI, RGRI, and RGBVI), achieving high accuracy in vegetation/non-vegetation discrimination (Kappa > 0.84) and overall classification accuracy exceeding 92.68%. Notably, VDVI demonstrated superior performance in distinguishing vegetation from non-vegetation, while crop coverage extraction showed the lowest segmentation error (0.63), highlighting its precision for structured plant communities. These findings expand the applicability of UAV remote sensing to biodiversity monitoring and ecosystem assessment. Future research could focus on refining the method for heterogeneous landscapes, exploring its scalability across seasons and regions, and integrating it with emerging sensor technologies to enhance ecological modeling and precision agriculture practices.

## Figures and Tables

**Table 1:** Visible light lens spectral range.

UAV Model	Red Band (nm)	Green Band (nm)	Blue Band (nm)	Source
DJI Mavic 2	620–670	520–600	450–520	Manufacturer Specs

**Table 2:** The confusion matrix of SVM-supervised classification results.

Land Cover	Wheat (Pixel)	Soil (Pixel)	Total Number of Samples	User Accuracy
Wheat (pixel)	29,611	140	29,751	0.9953
Soil (pixel)	157	22,405	22,562	0.9930
Total number of samples	29,768	22,545	52,313	
Producer’s accuracy	0.9947	0.9938		

**Table 3:** Classification performance (overall accuracy and Kappa coefficient) for distinguishing crops, trees, and shrubs from soil using four vegetation indices.

Vegetation Indices	Vegetation Types	Overall Accuracy	Kappa Coefficient
EXG	Crop	99.65%	0.99
	Soil		
	Tree	93.49%	0.85
	Soil		
	Shrub	99.83%	0.99
	Soil		
VDVI	Crop	99.78%	0.99
	Soil		
	Tree	94.22%	0.87
	Soil		
	Shrub	99.92%	0.99
	Soil		
RGRI	Crop	99.78%	0.99
	Soil		
	Tree	92.68%	0.84
	Soil		
	Shrub	98.71%	0.97
	Soil		
RGBVI	Crop	99.78%	0.99
	Soil		
	Tree	94.13%	0.87
	Soil		
	Shrub	99.93%	0.99
	Soil		

**Table 4:** Accuracy analysis of vegetation coverage in images with different resolutions (%).

Image Resolution (cm)	Vegetation Type	Threshold Extraction Result (%)	SVM (%)	Extraction Error (%)	Absolute Error	Standard Deviation
0.83	Crop	85.78	85.63	0.17	0.14	0.77
	Tree	74.90	76.57	−2.18	1.67	
	Shrub	62.18	62.94	−1.21	0.76	
1.25	Crop	85.66	85.63	0.03	0.02	1.20
	Tree	74.18	76.57	−3.13	2.40	
	Shrub	64.41	62.94	2.33	1.47	
1.67	Crop	84.57	85.63	−1.24	1.06	0.64
	Tree	75.97	76.57	−0.78	0.60	
	Shrub	64.81	62.94	2.96	1.87	
2.08	Crop	86.37	85.63	0.86	0.74	0.50
	Tree	75.34	76.57	−1.61	1.23	
	Shrub	64.67	62.94	2.75	1.73	

**Table 5:** Accuracy analysis of vegetation coverage in the validation area (%).

Vegetation Index	Vegetation Type	Threshold Extraction Result (%)	SVM (%)	Extraction Error (%)	Absolute Error	Standard Deviation
EXG	Crop	85.14	85.78	−0.74	0.63	0.60
	Tree	73.96	73.77	0.26	0.19	
	Shrub	61.28	60.02	2.10	1.26	
	Mixed	73.62	75.13	−2.01	1.51	
VDVI	Crop	84.91	85.78	−1.01	0.86	0.78
	Tree	73.85	73.77	0.12	0.08	
	Shrub	60.97	60.02	1.58	0.95	
	Mixed	77.11	75.13	2.64	1.98	
RGRI	Crop	86.92	85.78	1.34	1.15	1.48
	Tree	74.55	73.77	1.06	0.78	
	Shrub	57.52	60.02	−4.17	2.50	
	Mixed	71.08	75.13	−5.38	4.04	
RGBVI	Crop	87.46	85.78	1.96	1.68	1.10
	Tree	75.90	73.77	2.90	2.14	
	Shrub	59.11	60.02	−1.53	0.92	
	Mixed	71.58	75.13	−4.72	3.55	

## Data Availability

Data will be made available upon request.
